# Online comic-based art workshops as an innovative patient and public involvement and engagement approach for people with chronic breathlessness

**DOI:** 10.1186/s40900-023-00423-8

**Published:** 2023-03-30

**Authors:** Samantha L. Harrison, Julian Lawrence, Sophie Suri, Tim Rapley, Kirsti Loughran, James Edwards, Louise Roberts, Denis Martin, Joanne E. Lally

**Affiliations:** 1 School of Health and Life Sciences, Teesside University, Borough Road, Middlesbrough, TS1 3BA UK; 2 Department of Social Work, Education and Community Wellbeing, Northumbria University, Coach Lane Campus West, Newcastle Upon Tyne, NE7 7XA UK; 3 Breathe Easy Darlington, Darlington, UK; 4 Population Health Sciences Institute, Faculty of Medical Sciences, Newcastle University, Baddiley-Clark Building, Richardson Road, Newcastle Upon Tyne, NE2 4AX UK

**Keywords:** Breathlessness, Cartooning, Patient and public involvement and engagement

## Abstract

**Background:**

Talking about breathlessness can be emotionally challenging. People can feel a sense of illegitimacy and discomfort in some research contexts. Comic-based illustration (cartooning) offers an opportunity to communicate in a more creative and inclusive way. We used cartooning in patient and public involvement and engagement (PPIE) work to explore symptoms of breathlessness and their impact on peoples’ everyday lives.

**Main body:**

Five, 90-min cartooning workshops were delivered online to members of Breathe Easy Darlington (UK). The workshop series involved 5–10 Breathe Easy members and were facilitated by a professional cartoonist supported by three researchers. The experience of living with breathlessness was represented via illustrations of cartoon characters and ideas explored in subsequent conversations. Cartooning was fun and the majority found it a nostalgic experience. Sharing the experience helped the research team develop new understandings of breathlessness and fostered relationships with the Breathe Easy members. The illustrations showed characters leaning against objects, sweating and sitting down, demonstrating living with the sensation of not being in control.

**Conclusion:**

Comic-based art, as a fun and innovative PPIE approach. It facilitated the research team becoming embedded in an existing group who will act as PPIE members on a long-term research programme. Illustrations enabled storytelling and fostered novel insights into the lived experiences of people with breathlessness including sensations of a loss of control, disorientation, and unsteadiness. This will impact on work investigating balance in people with chronic obstructive pulmonary disease. This model has potential to be applied in a range of PPIE and research contexts.

## Abstract (summary)

Talking about breathlessness can be difficult and cause feelings of anxiety. Involving people with breathlessness in research activities can also be challenging. Research contexts can make people feel uncomfortable as they may struggle to understand and feel like they don’t belong. Comic-based illustration (cartooning) offers an opportunity to communicate in a different way. Cartooning was used with members of Breathe Easy Darlington, a support group for people with breathlessness, as a way of helping the research team understand how breathlessness impacts daily lives. Five 90-min cartooning workshops were delivered online and involved 5–10 Breathe Easy members per session. The sessions were led by a professional cartoonist whilst the research team facilitated discussions about breathlessness and related issues. The experience of living with breathlessness was illustrated via cartoon characters and ideas were explored through conversations about the illustrations. People found cartooning fun and sharing the experience with the research team helped them to become part of an existing group who will support a long-term research project directly impacting research investigating balance in people with lung disease. The Breathe Easy members were able to tell their stories via illustrations allowing the research team an insight into different aspects of living with breathlessness. The illustrations showed characters leaning against trees and chairs, sweating and sitting down. The need for physical support caused embarrassment, while people struggled to appear “normal”. Conversations about the illustrations revealed that the idea of “balance” aligns with a loss of control, disorientation, and unsteadiness which causes intense fear and shame.

## Background

### Why are innovative patient and public involvement and engagement (PPIE) activities needed for people with chronic breathlessness?

Patients often find breathlessness difficult to describe and the invisibility of a chronic lung condition means people often don’t feel believed [1]. In the research teams’ clinical experience and conversations with many individuals who experience chronic breathlessness, emotive imagery is used to describe the seriousness of their situation and to bolster legitimacy of symptoms. Examples include images such as lungs filling with water, trying to breathe through a straw and/or a sense of having weights on your chest. Communicating the sensation of breathlessness can also be emotionally challenging, provoking anxiety which in turn increases shortness of breath. This poses another barrier to verbal communication as breathlessness can make it difficult to talk. People with chronic breathlessness tend to be older adults (aged > 70 years) who may be experiencing cognitive decline as part of the aging process and accelerated by the presence of a progressive lung condition [2]. Coupled with this is the fact that individuals with lung conditions tend to come from low-socioeconomic groups and can feel marginalised in their interactions with researchers [3, 4]. As a result, people with chronic breathlessness may struggle to convey the complexity and seriousness of their symptoms, meaning patient narratives, gained through traditional research methods (e.g., interviews), could fail to fully express the impact of symptoms upon everyday lives.

Engaging people with chronic breathlessness in research activities can be tricky but taking a more innovative and creative approach can offer an opportunity to communicate in a different way that is potentially more inclusive. This is important because increasing the diversity of the people involved in research likely increases the involvement of those who the research affects leading to greater impact [5]. Arts in health activities, such as dance, singing and music, are gaining popularity in respiratory medicine and evidence is building to support such activities as ways to improve mood, physical activity and body awareness [6–9]. Dance as an activity also appears to be well accepted with one study reporting approximately 50% uptake and 87% completion rate [7]. Furthermore, qualitative findings report dance and playing the harmonica to be enjoyable and fun activities that might be sustained [8, 9]. However, arts-based activities such as drawing and illustration may also have an important role in facilitating understandings of symptoms and the day-to-day difficulties faced by people with breathlessness.

### Why use comics/cartoon illustration as a PPIE approach?

Comic-based art activities, such as illustration and cartooning, have been employed in educational settings since Swedish schoolteacher Rodolph Töpffer developed the medium as a classroom teaching tool in 1823 [10, 11]. Over the last 200 years, comics and cartooning have been used by education professionals as techniques for negotiating identity, as a research methodology, and as academic outputs [12–16]. Drawing and cartooning have been used with a range of groups to draw out new and insightful knowledge about the illness experience. For example, graphic medicine brings together cartooning and healthcare by *“capturing the illness experience in the comics medium. Established definitively as a field in 2007… graphic medicine refers to the comics' distinct engagement with and performance of illness”* [17].

In one research project, the emotional burden of asthma was expressed powerfully via drawings [18] and pain cognitions and barriers to recovery have been illustrated in another [19]. Art has also been used to help people with obsessive compulsive disorder (OCD) express what it is like to live with the debilitating condition [20] and people with Chronic Obstructive Pulmonary Disease (COPD), when asked to draw their lungs, did so with medical images (e.g. X-rays) showing the impact medical technologies and clinic visits have on embodiment [21].

Cartoon illustration (or comics/cartooning) is a type of drawing tool that is growing in popularity in health communication. Drawing comics allows for abstract entities and exaggeration in a series of images that promote storytelling. Comics and cartooning perform stories and storytelling through what McCloud [22] describes as the medium’s *“(j)uxtaposed pictorial and other images in deliberate sequence, intended to convey information and/or to produce an aesthetic response in the viewer*”. In this way, telling stories with original comics and cartoons is a multimodal motor skill that triangulates the drawing system, the perceptual system, and meaning [23].

This *“cognitive model of drawing”* (Cohn [23], 172) opens new areas of exploration for the artist. For instance, cognitive links to childhood memories of reading comics and drawing evoke generally positive reactions in many people. Groensteen writes, *“comics still have a privileged relationship with childhood because it is in childhood that each of us discovered them and learnt to love them”* [10]. This is important because people are more likely to engage in an activity that they enjoy and can be associated with positive experiences.

This article reports the use of comic-based illustration workshops as a PPIE activity to explore symptoms of breathlessness and their impact on the everyday lives of people living with chronic breathlessness. The activity was conducted in the context of a research program focused on understanding and addressing balance problems in people with COPD, more details about the project can be found at: B-PuRe: Reducing the risk of falls in people with COPD—ARC (https://arc-nenc.nihr.ac.uk/projects/b-pure/). PPIE is defined as *“research being carried out with or by members of the public rather than to” *and is now central to shaping the research of those applying for funding in the UK to conduct health research [24, 25]. Including the *“patient voice”* is seen as improving the quality and relevance of research and the National Institute of Health and Social Care Research (NIHR) recommends that the lived experience of people be understood to refine research questions [26, 27]. The PPIE approach we undertook was underpinned by the national standards for public involvement to ensure diversity, inclusion and quality [28]. We drew on the methods of cartooning to introduce a program of work, foster relationships and facilitate discussion around a sensitive subject in a non-direct way.

## Main text

### The research teams’ approach

Comic-based illustration workshops were offered to members of Breathe Easy Darlington (UK), a support group for people with lung disease and breathing problems. The workshops were part of the PPIE activities planned for a large NIHR funded programme of work focused on investigating balance in people with COPD and as such ethical approval was not required.

The series consisted of five, 90-min workshops, delivered online via Zoom between April and September 2021 and were facilitated by a professional cartoonist and educator specialising in comic books (JL). The purpose of the workshops was to teach cartoon illustration, with the aim to understand the experiences of living with chronic breathlessness. SH and/or KL (academics and physiotherapists experienced in the care and research of people with chronic breathlessness) and JEL (an expert in innovative approaches to PPIE including arts-based) were active participants in the workshops. Conversations and discussion were encouraged, and these manifested in two ways: either through oral conversations over Zoom or as typed messages in the chat. Fieldnotes were taken and alongside the participants cartoon drawings these were used to shape the research teams understandings about pertinent issues including the sensation of breathlessness linked to control and emotions. Meetings between SH and JEL were held after each workshop to synthesis the main issues. The three researchers agreed the lessons learned during two meetings and subsequent emails. The PPIE work, would in turn help refine the research questions, methods and public involvement of the larger research programme.

**Fig. 1:**
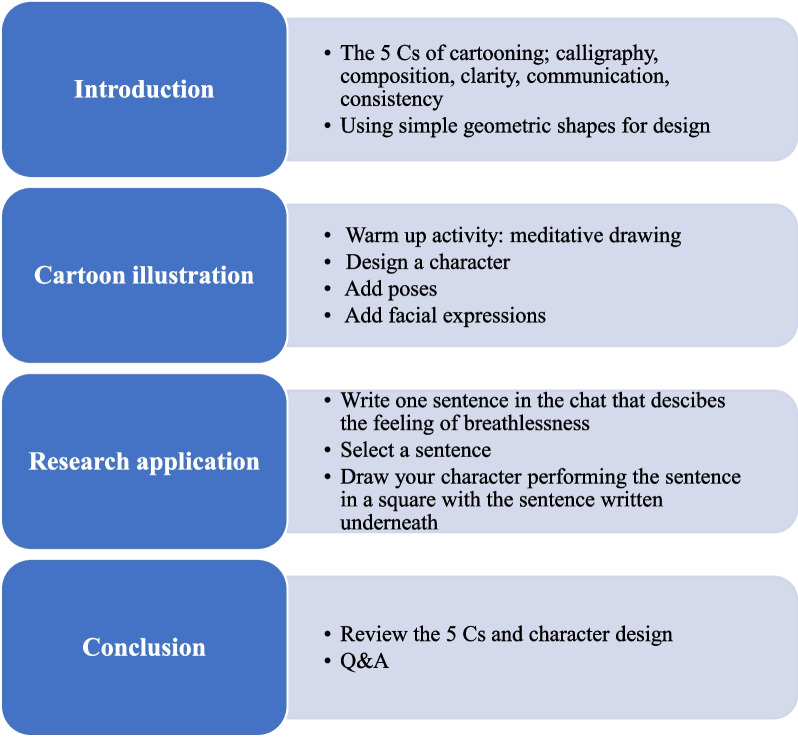
Example of content from the first cartoon-illustration workshop

The first workshop served as an introduction (Fig. 1). Cartoonist Ivan Brunetti [29] posits the 5 Cs of Cartooning as essential prerequisites for making comics, and a discussion regarding their importance was facilitated. The 5 Cs are:

- Calligraphy
- Composition
- Consistency
- Clarity
- Communication

*Calligraphy* refers to the participant’s drawing style. Elements of *composition* were demonstrated by exploring various camera angles such as long shots and close ups. *Consistency* was maintained by employing simple geometric shapes as the foundation of the character designs. In this way, the character drawn by the participant is easily reproduced and recognised from one panel to the next. *Clarity* was encouraged through the use of exaggerated poses and facial expressions. Thus, strong *communication* through comics can be successfully achieved by adhering to the first 4 Cs of Cartooning.

**Fig. 2:**
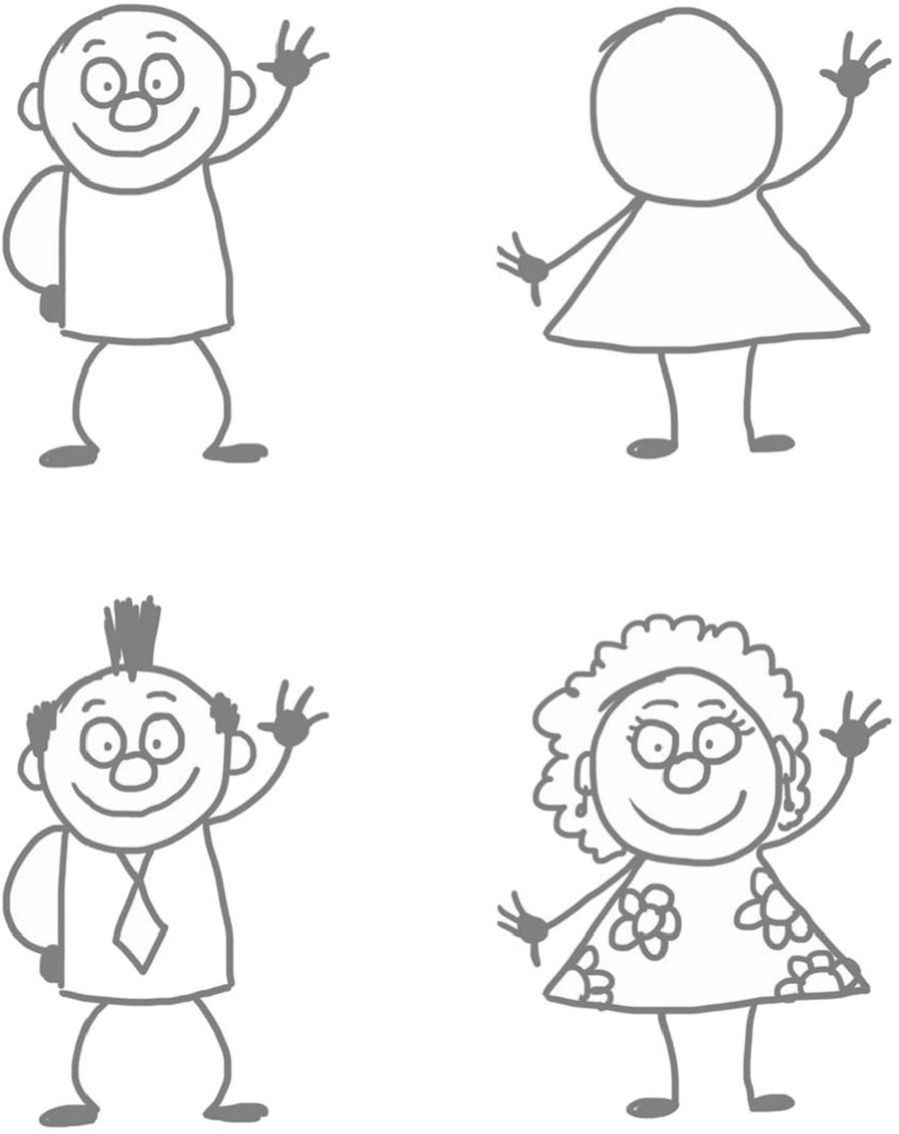
Simple character designs participants can customise

The form of cartoon drawings used basic geometric shapes such as circles, squares, ovals, triangles, etc. to construct uncomplicated cartoon characters. The simplicity of the designs permitted participants to customise and create their own original characters by adding features such as hair and clothes (Fig. 2). Participants were encouraged to write one sentence in the chat to describe their feelings of breathlessness. We all took a moment to quietly read through the responses. Participants were asked to:

- select one of the sentences (it could be their own or one from another participant)
- draw a large square in the middle of a sheet of A4 paper
- write the selected sentence from the chat under the square
- draw their character performing the sentence inside the square.

In this way, a single panel “gag” cartoon is created.

The content from the five workshops is displayed in Table 1. Participants were also encouraged to draw in their own time between workshops, developing their characters and skills. However, few did draw cartoons alone, preferring instead to have guidance from the cartoonist and finding it to be a more fun activity done in a group setting. This might have changed as the participants confidence in their drawing abilities increased.

**Table 1:** The content of the five workshops

Date	Warm-up	Activity 1	Activity 2	Next steps
Workshop 1: April 7, 2021	Draw a mindful spiral	How to design a cartoon character	Brainstorm logo design ideas	Discovering comics and cartooning
Workshop 2: May 19, 2021	5 Cs of Cartooning/Draw a car	Simple geometric shapes/Review drawing character	Draw a single-panel gag cartoon	Explore single-panel cartoons (Private Eye, New Yorker)
Workshop 3: June 16, 2021	Discuss drawing in 3-D	Two-panel exercise	Two-panel exercise, cont’d	Sharing two-panel cartoons with the group
Workshop 4: July 21, 2021	Speed drawing exercise	Facial expressions in comics	Camera angles in comics	Share 4-panel strips with group
Workshop 5: August 18, 2021	Review previous topics: geometric shapes, 3-D, 5 Cs of Cartooning	Group discussion of comics such as Beano, Dandy	Create a 4-panel comic strip with camera angles	Conclusion and wrapping up

### Lessons learned

#### Comic-based illustration is a fun and, for most, nostalgic activity

For many people memories of childhood experiences with comics in the past link to drawing comics in the present [30]. For example, one member reminisced watching the animated cartoon *“Bod and the Apple”* as a child, and another was reminded of the sound and feel of a pencil “*scratching*” across the paper. One person did not find the nostalgia of the activity enjoyable because comics and drawing remined him of times when he was unwell and confined to bed.

#### Artistic skill is not important

Some people expressed concern regarding their artistic abilities, or lack thereof. It was, therefore, crucial for everyone to be re-assured from the outset *“that artistic skill (is) not important”* and that the focus was on perceptions and experiences of breathlessness [18]. As the workshops progressed, Breathe Easy members confidence and trust increased, and they were not shy to laugh at and gently tease each other and the researchers about their artistic efforts. Thus, a friendly on-line community developed whereby participants were happy to share their cartoons with the group either by holding them up to the screen or taking pictures and sending their drawings over the chat or through email.

#### Sharing the experience and finding it challenging, regardless of role, helped to develop bonds

**Fig. 3:**
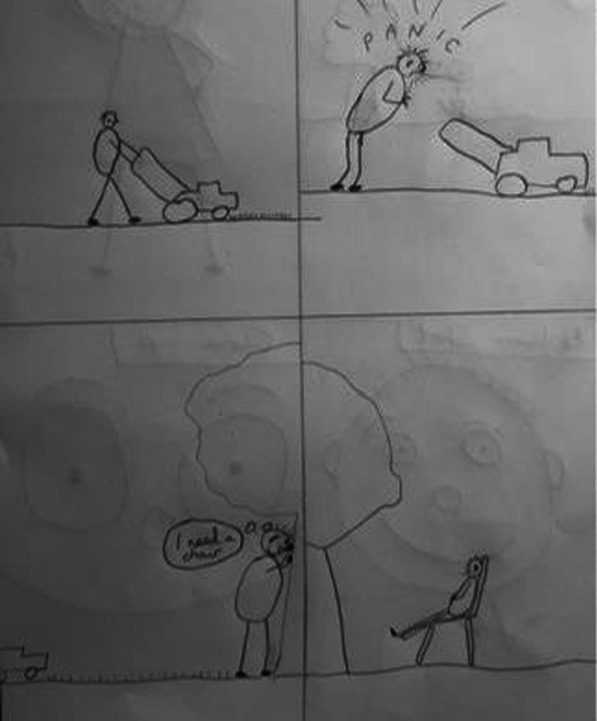
Example illustrations by a Breathe Easy member leaning on a tree (shared with permission)

Online meetings can be awkward, and conversation stunted, however the task of drawing acted as a *“social lubricant”* and the group bonded over a shared experience. Through participation in the activity a mutual trusting relationship between Breathe Easy members and the researchers developed, that would help support their involvement in ongoing research. The members agreed “*you (researchers) are one of us now”* (extract from fieldnotes). Learning about the lived experience was driven by the members; their own illustrations directed the discussion topics rather than being driven by researcher-led questioning. This, in turn, helped to build trust and explore areas of interest to the members. Engaging in informal discussion allowed for a far more organic emergence of lived experience, with the opportunity for the research team to instantaneously check meaning by clarifying interpretation of illustrations with the authors’ intensions of the image. For example, e.g. *“so… this person mowing the lawn is leaning against a tree not just to recover their breathing but to feel steady?”* (Figs. 3).

#### Breathlessness is a whole-body sensation contributing to a loss of control

**Fig. 4:**
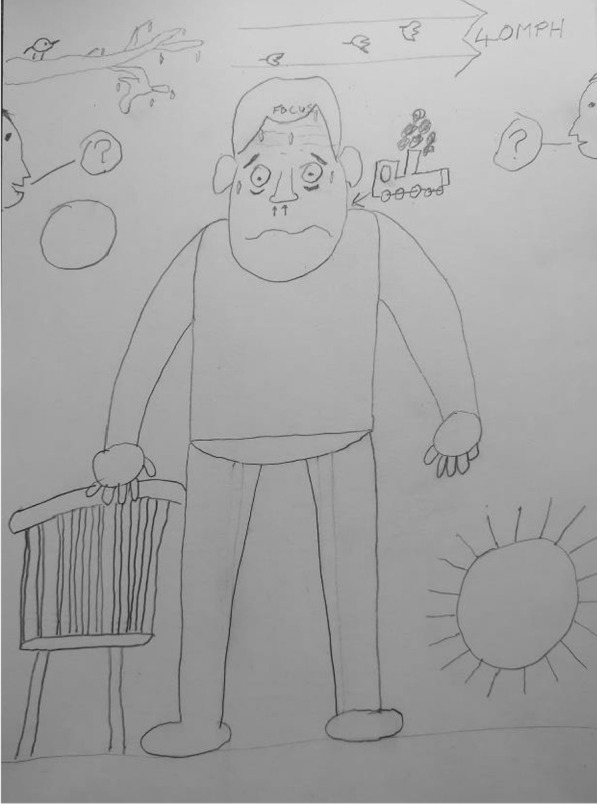
Example illustration by a Breathe Easy member holding onto a chair (shared with permission)

**Fig. 5:**
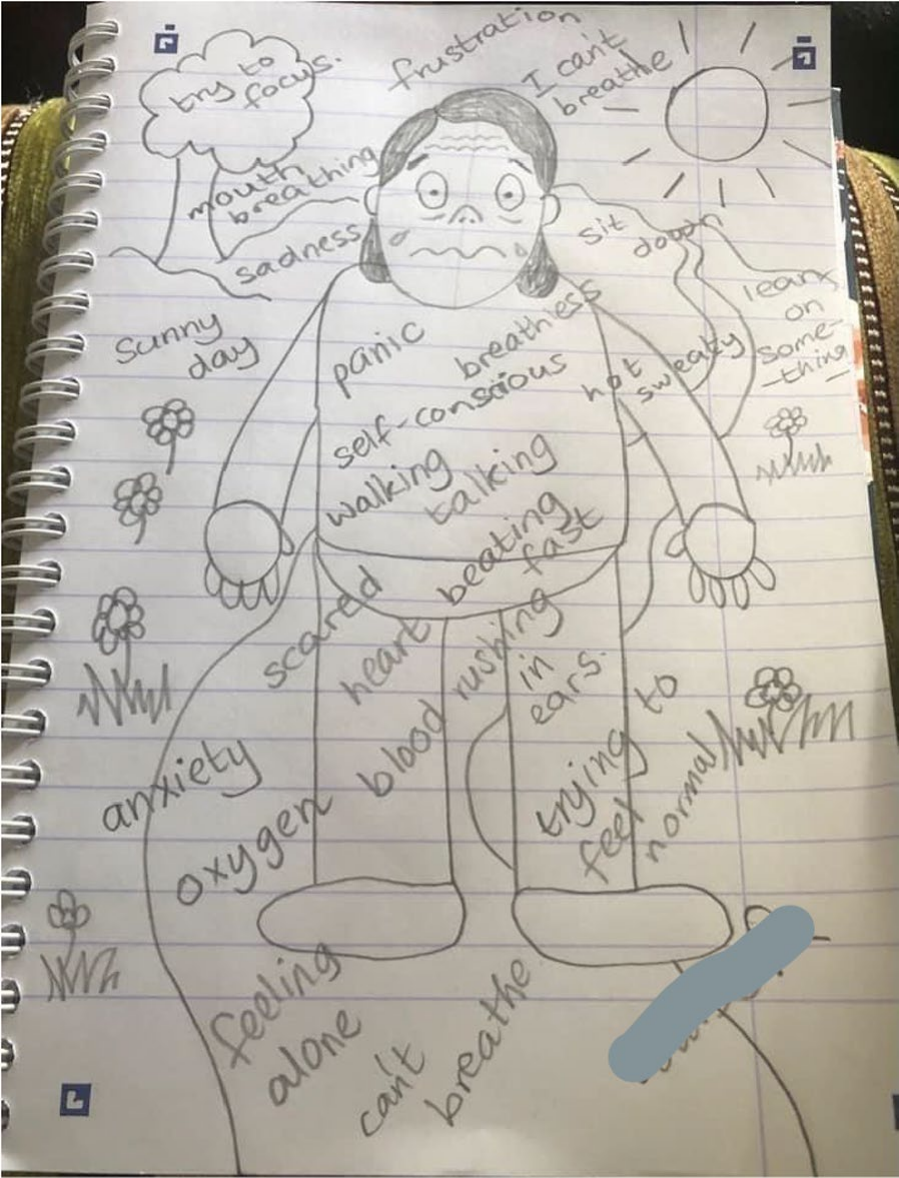
Example illustration by a Breathe Easy member sweating and with annotations (shared with permission)

**Fig. 6:**
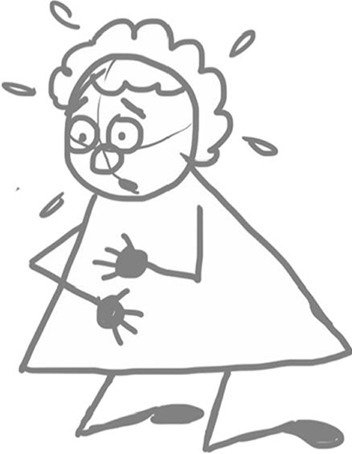
Example illustration by JL of a breathless character kneeling

The Breathe Easy members’ intense feelings of breathlessness were demonstrated by the visual metaphors in their comic-based drawings. For instance one participant likened his breathing to a *“chuffing train”* (Fig. 4) and by drawing postural changes such as shoulders being up by their ears (Figs. 4 and 5). Accounts of breathlessness were interwoven with an overwhelming sense of losing control and feeling unsteady which went beyond individuals’ cognisance. Breathe Easy members drew their characters sitting down, leaning or holding onto an object for support and to recover their breathing. Another example is observed in Figs. 4, 5 and 6 whereby cartoon characters are sitting, kneeling or holding onto a chair. In conversation, participants said they felt shaky and they reached for something to make them feel safe, otherwise they might fall over. Figure 4 shows two people either side of the page with question marks in speech bubbles. When asked what this meant the participants described a *“brain/body divide”*, an inability to concentrate and feelings of confusion. They laughed at themselves and recounted times they forgot things. One lady recalled how she would walk upstairs which would make her breathless and forget what she came up for. Sharing experiences and finding out that others experienced similar sensations was consoling.

#### Breathlessness is frightening and cyclical

**Fig. 7:**
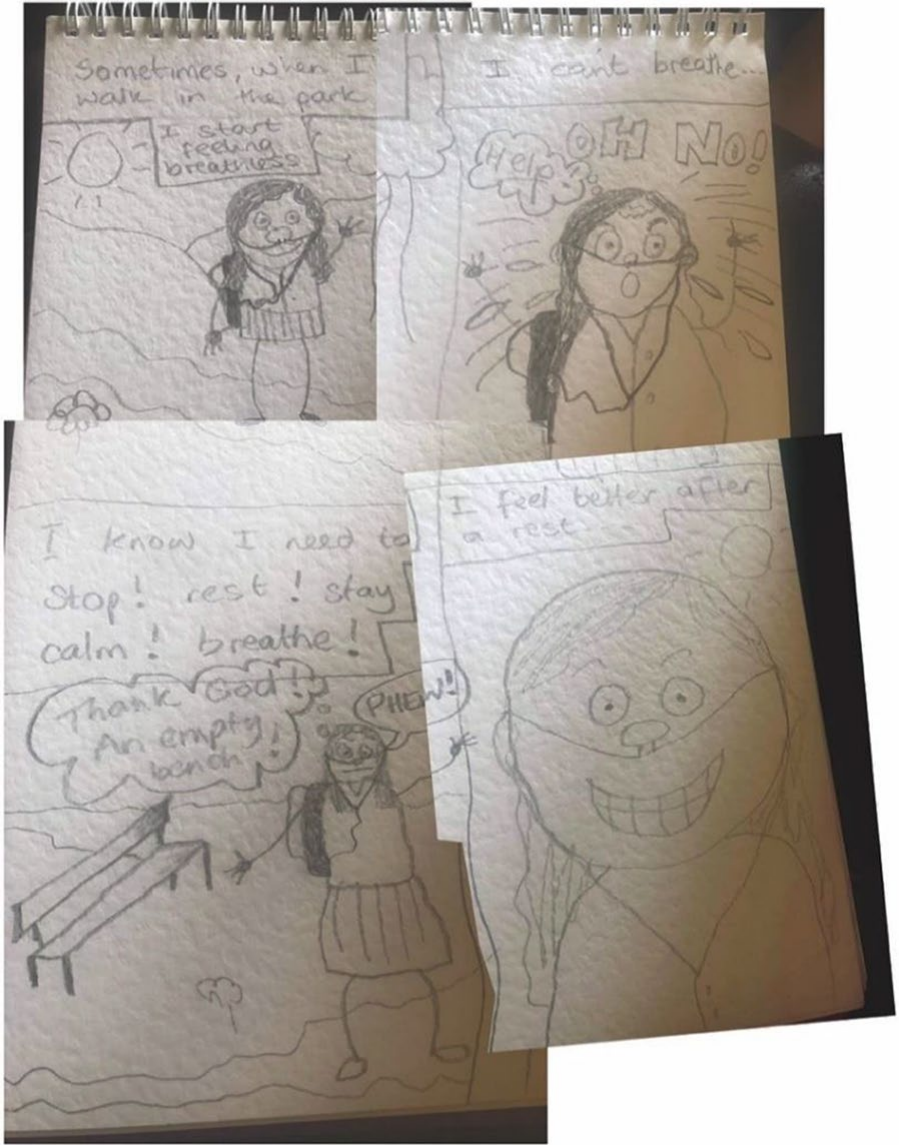
A four-panel comic describes one person’s experience of breathlessness (shared with permission)

Breathe Easy members’ characters looked unwell, for example they were sweating (Figs. 4 and 5) which is also consistent with a fear response. The hand-drawn facial expressions (eyes popping, beads of sweat etc.) on their characters and the annotated images (Fig. 5) emphasised intense emotions in response to breathlessness (e.g. scared, anxious, confused, angry and frustrated). However, by reaching for something (such as a tree or the back of a chair (see Figs. 3 and 4) breathing could be recovered and a feeling of control resumed. This was expressed as a relief by the Breathe Easy members, who were able to reflect on the activity and view this as an achievement. In Fig. 7, the final panel shows a character with a big grin after resting on a bench.

#### It is necessary to reframe the idea of “balance”

The stories that were told during the workshops did not refer to *“balance”* specifically, instead, the participants recounted times they felt unsteady, stumbled, or felt disoriented. For example, one gentleman recounted a time he fell backwards into a bush when playing with grandchildren, it was told in a humorous way and made the group laugh but he recounted feeling disoriented and unsteady. Another member described herself as *“looking like she’d had a few [alcoholic drinks]”* staggering down steps holding onto the rail and said this made her feel embarrassed. As these discussions about breathlessness emerged, researchers reflected on these stories and reframed their ideas about balance in the intended research as a result, seeing balance as tied to broader ideas around loss of control.

Table 2 displays the ways in which this PPIE approach maps onto the national standards.

**Table 2:** The innovative PPIE approach mapped onto UK standards for public involvement and engagement with consideration of areas for improvement

UK standard	Summary of standard	Activity—how the standard was met	Areas for improvement
Inclusive opportunities	Offering public involvement opportunities that are accessible and reach people and groups according to research needs	Comic-based illustrations to describe complex and emotive symptoms in those who may find verbal communication difficult due to feelings of illegitimacy, breathlessness, cognitive issues, feelings of marginalisation	Due to COVID-19 the workshops were delivered online and therefore only accessible to those with access to a computer and IT skills. However, the workshops could be delivered face-to-face. Future work needs to include a more diverse population in terms of class and ethnicity (all group members were white)
Working together	Work together in a way that values all contributions, and that builds and sustains mutually respectful and productive relationships	Recognising the role of the participants in shaping the research programme and developing the logo demonstrates value	Consider how the researcher being active participants impacted the outcomes
		The researchers were active participants in the workshops. Sharing the experience helps to foster trust and build relationships	Make the purpose of the workshops very clear (e.g., give an example of how this type of activity has shaped other research programmes)
Support and learning	Offer and promote support and learning opportunities that build confidence and skills for public involvement in research	PPI through a shared experience which is enjoyable is less intimidating and provides an example of a PPI activity	Ask participants to evaluate the workshops formally and take this feedback forward to other PPI activities
		Sharing illustrations and experiences helps build confidence and relationships	
Communication	Use plain language for well-timed and relevant communications as part of involvement plans and activities	Use of non-verbal communication, namely comics, promotes imagination and storytelling, facilitating ‘meaning-making’	People may have been less open on an online platform —create a space for people to disclose information privately and on reflection
			To develop a communication plan with the PPI contributors to consider modes of communication and content
Impact	Seek improvement by identifying and sharing the difference that public involvement makes to research	Communicating the impact of their contributions to the workshop participants and the wider Breathe Easy group members. Writing this up as a academic paper. Presenting drawings as medical graphics and at an art event	Gather participants reflections on the impact the PPI activity has had
		Informs project focus (reframing balance) to loss of control regaining steadiness. The logo offers a tangible demonstration of their input	
Governance	Involve the public in research management, regulation, leadership and decision making	Setting ground rules to respect everyone’s voice and to keep shared experiences confidential	Increase involvement in the planning stage for PPI involvement and the types of activities
		Respecting confidentiality when embedding descriptions into written publications and reports	

### Future directions

Since the comic-based illustration workshops took place, the research team have become embedded within the Breathe Easy community. The active participation of researchers in the workshops and other activities has helped to foster trusting relationships. The research team have since attended social events, supported fund raising efforts, and organised and joined in other new collective activities (e.g. yoga, dance, walking football) that have prompted feelings of nostalgia and facilitated new discussion about the lived experience of breathlessness. The importance of building meaningful relationships with communities is underpinned by the Community Engagement Toolkit’s ten guiding principles [31]. Continuity in PPIE membership throughout the duration of a research programme is also advantageous to the research team as there is an initial investment in training and collaborative partnerships take time to build. By becoming embedded into an existing group researchers can have access to a wider and more diverse population, hopefully leading to the research produced being more inclusive. Individuals are driven to join a support group to share illness experiences, rather than to bring about change meaning they are probably a more diverse group of individuals than those who self-select to be part of a research steering committee [32, 33].

The workshops have prompted the research team to reframe the way they think about balance. They have considered what balance means to people with chronic breathlessness and understand that this differs considerably to what health care professionals are referring to when they talk about having *“poor balance”* or recommending balance training. In a follow up activity, the research team asked Breathe Easy members to write one word that they think of in response to the word *“balance”*. Responses ranged from happiness and wellbeing to dizziness, control and concentration. Reflecting on balance as a whole-body sensation that is inexplicably linked to breathlessness, which is accompanied by an overwhelming sense of losing control along with being unsteady and disorientated is a novel way of thinking for the research team. It will shape the way they describe the purpose of the research programme in patient documentation and the way a subsequent balance training intervention is packaged, so it is potentially more meaningful for individuals with breathlessness.

Notably, this manuscript reports the use of comic-based art as a PPIE approach. However, the lessons learned could be transferred to be used as a research method, as part of an interview or focus group based study, as well as part of an ethnographic study, to enable additional insight into an emotive issue, and to understand the lived lives of individuals with chronic conditions. Often, very core issues can become routine to the patient, seen but unnoticed, to those living with a chronic illness and are therefore not discussed with researchers or healthcare professionals. Illustrations can allow conversations to be shaped in different ways (e.g. the participants’ comic-based drawings), to stimulate, new, organic, discussions about taken for granted issues that become important when living with a debilitating condition.

## Conclusion

Using comic-based art as an innovative and fun PPIE approach has facilitated the research team to become embedded into an existing group who will act as PPIE members on a long-term programme of research. Sharing an experience—in this case cartoon illustration—has helped foster trusting relationships between PPIE members and the research team that are likely to be sustained. Cartoon illustrations have enabled storytelling and directed topics for discussions that have prompted the research team to reassess their assumptions about *“balance”,* as something consisting of strength, co-ordination and even weight distribution and what it means to people with breathlessness. Findings will feed into the substantive study, aid communication with participants, shape how we approach PPIE work in future and, we believe, increase inclusivity. This is an enjoyable shared experience that could be applied as a useful approach to a range of PPIE and research contexts.

## Data Availability

Not applicable.

## Abbreviations

PPIE: Patient and public involvement and engagement
OCD: Obsessive compulsive disorder
COPD: Chronic obstructive pulmonary disease
NIHR: National Institute for Health and Social Care
